# The power of the group: comparison of interviews and group concept mapping for identifying patient-important outcomes of care

**DOI:** 10.1186/s12874-018-0656-x

**Published:** 2019-01-08

**Authors:** Kristin L. Rising, Marianna LaNoue, Alexzandra T. Gentsch, Amanda M. B. Doty, Amy Cunningham, Brendan G. Carr, Judd E. Hollander, Lori Latimer, Larry Loebell, Gail Weingarten, Neva White, Geoffrey Mills

**Affiliations:** 1 Department of Emergency Medicine, Thomas Jefferson University, 1025 Walnut St, Suite 300, Philadelphia, PA 19107 USA; 2 College of Population Health, Thomas Jefferson University, Philadelphia, PA USA; 3 School of Medicine, Department of Family and Community Medicine, Thomas Jefferson University, Philadelphia, PA USA; 4 Center for Urban Health, Thomas Jefferson University, Philadelphia, PA USA; 5 Philadelphia, PA USA

**Keywords:** Group concept mapping, Interviews, Patient-centered outcomes, Outcome elicitation, Patient engagement, Brainstorming

## Abstract

**Background:**

Data are limited regarding how to effectively and efficiently identify patient priorities for research or clinical care. Our goal was to compare the comprehensiveness and efficiency of group concept mapping (GCM), a group participatory method, to interviews for identifying patient goals when seeking care.

**Methods:**

We engaged patients with moderately- to poorly-controlled diabetes mellitus in either GCM or an individual interview. The primary outcome was the comprehensiveness of GCM brainstorming (the first stage of GCM) as compared to interviews for eliciting patient-important outcomes (PIOs) related to seeking care. Secondary outcomes included 1) comprehensiveness of GCM brainstorming and interviews compared to a master list of PIOs and 2) efficiency of GCM brainstorming, the entire GCM process and interviews.

**Results:**

We engaged 89 interview participants and 52 GCM participants (across 3 iterations of GCM) to identify outcomes most important to patients when making decisions related to diabetes management. We identified 26 PIOs in interviews, 33 PIOs in the first GCM brainstorming session, and 38 PIOs across all three GCM brainstorming sessions. The initial GCM brainstorming session identified 77% (20/26) of interview PIOs, and all 3 GCM brainstorming sessions combined identified 88% (23/26). When comparing GCM brainstorming and interviews to the master list of PIOs, the initial GCM brainstorming sessions identified 80% (33/41), all 3 GCM brainstorming sessions identified 93% (38/41) and interviews identified 63% (26/41) of all PIOs. Compared to interviews, GCM brainstorming required less research team time, more patient time, and had a lowest cost. The entire GCM process still required less research team time than interviews, though required more patient time and had a higher cost than interviews.

**Conclusions:**

GCM brainstorming is a powerful tool for effectively and efficiently identifying PIOs in certain scenarios, though it does not provide the breadth and depth of individual interviews or the higher level conceptual organization of the complete process of GCM. Selection of the optimal method for patient engagement should include consideration of multiple factors including depth of patient input desired, research team expertise, resources, and the population to be engaged.

**Trial registration:**

Registered on ClinicalTrials.gov, NCT02792777. Registration information submitted 6/2/2016, with the registration first posted on the ClinicalTrials.gov website 6/8/2016. Data collection began on 4/29/2016.

**Electronic supplementary material:**

The online version of this article (10.1186/s12874-018-0656-x) contains supplementary material, which is available to authorized users.

## Background

Despite national focus on the importance of patient-centered research and clinical care delivery, many researchers and clinicians use endpoints that they presume to be important to patients rather than directly engaging patients to identify patient-important outcomes (PIOs). PIOs are outcomes that take into account individual patient values and preferences [1–3]. While patient-reported outcomes (PROs) are often used as PIOs, PROs and PIOs are distinct concepts. With PROs, the measurements come directly from the patients, regardless of whether the outcomes being measured are important to patients [4, 5]. In contrast, PIOs are grounded in patient values and preferences [1–3]. While PIOs are often also PROs, this is not always the case (eg. mortality). To date, there is no formal guidance on how to best identify PIOs for use in research.

The definition of PIOs suggests the need to have direct patient input for identification of PIOs. While identification of PIOs does not always involve measure development, best practices for engaging patients to develop patient-centered measures can inform approaches for PIO identification. Patient-centered measure development starts with a content elicitation phase, in which the population to which the measure would apply is engaged to identify relevant content related to the topic of interest [6, 7]. After content elicitation, participant-level data then need to be organized into higher level domains to develop an overall conceptual framework and identify meaningful and more broadly applicable outcomes or measures. Historically, interviews and focus groups have been the standard approaches to engaging populations of interest for content elicitation, with researchers then organizing the participant-level data into higher level domains [6–8]. Interviews allow in-depth exploration of topics, and are particularly suited to sensitive topics that individuals may not want to explore in a group. However, interviews are resource-intensive to conduct and analyze; thus, they have limited scalability [9]. Focus groups – which can be thought of as group interviews – offer a more efficient means of engaging individuals. Focus groups encourage participant interaction to collectively explore, clarify, and build upon one another’s ideas, thereby harnessing the power of the group to quickly elicit a range of perspectives. However, focus groups require skilled facilitation to avoid domination by certain individuals or “group think,” and are also resource-intensive to conduct and analyze [10, 11].

Group concept mapping (GCM) is a mixed methods approach to patient engagement used widely across disciplines for program planning and evaluation [12–21] that addresses the primary time and resource limitations of interviews and focus groups. In addition to facilitating content elicitation, GCM also engages participants to do the next step of data analysis in which individual ideas are organized into higher domains, thus eliminating a significant portion of researcher bias in the data analysis phase. In GCM, participants are asked to 1) brainstorm ideas in response to a prompt or question, 2) sort ideas into conceptually-similar piles and 3) rate ideas along predefined dimensions (e.g. importance, feasibility). The result of this process is a “concept map” that displays all the brainstormed ideas aggregated into clusters that portray similarly-sorted ideas in higher-level domains. As a final step, participants are asked to interpret and refine the “map”, including naming of all the clusters [22]. GCM has been used by some as an alternative means for both content elicitation and data analysis for measure development [23–27].

Though GCM offers promise as an effective and more efficient means of content elicitation and idea organization, no studies have been performed to assess both the comprehensiveness of the content elicitation phase of GCM and the efficiency of GCM compared to traditionally-accepted methods. Our goal with this work was 1) to compare the comprehensiveness of the brainstorming phase of GCM to interviews for content elicitation and 2) to compare the efficiency of GCM (both the initial brainstorming phase as well as the entire process) and individual interviews when engaging patients with moderately- to poorly-controlled diabetes mellitus (DM) to identify PIOs for diabetes care. We hypothesized that the breadth and content of data produced by GCM brainstorming and one-on-one interviews would be similar (comprehensiveness). In addition, we hypothesized that the time and personnel resources required to complete just content elicitation (GCM brainstorming) as well as content elicitation along with data analysis (entire GCM process) would be less than those required for interviews (efficiency). There was no measure of success being tested with this work, rather we strove to generate data regarding the output and resource intensiveness of each method to inform future selection of methods for participant engagement. With this work, we aim to advance the field of patient-centered research by providing researchers with data regarding the most scalable, efficient, and nimble method to engage patients in defining truly patient-important outcomes.

## Methods

### Overall approach

We employed a mixed-methods and participatory approach in this study. This work was performed in close collaboration with a patient advisory board, the Patient Advocate and Key Stakeholders Advisory Board (PAKSAB). The research team met with the entire PAKSAB quarterly from project inception to completion and three PAKSAB members served as part of the research team to assist with patient recruitment, data collection, data analysis and interpretation. All reference to the “research team” that follows includes these 3 PAKSAB members.

### Study setting and participants

Testing these two methods of patient engagement required the selection of a uniform patient population. Despite the availability of evidence-based guidance and treatments (pharmacologic and non-pharmacologic) for diabetes mellitus (DM), many patients with DM do not achieve optimal glycemic control [28], putting them at higher risk of complications and increased healthcare utilization [29]. We believe that this lack of achieving DM control despite available and proven effective treatments suggests a disconnect between medically-identified and patient-identified priorities. Thus, we decided to engage patients with moderately- to poorly-controlled diabetes mellitus (DM), as we thought that the output generated from this study – a list of PIOs from these patients who have inadequately controlled diabetes – has potential to inform a more effective approach to caring for this vulnerable population.

We recruited patients with moderately- to poorly-controlled DM from a single large urban academic healthcare system located in Philadelphia, PA. We included adult patients (age 18 and older) who were English speaking, had a history of DM type 1 or type 2 and were able to provide informed consent. Patients were interviewed during an ED visit (acute care), within 7 days of discharge from the inpatient internal medicine or family medicine services (post-acute care), or immediately before or after a routinely scheduled primary care visit within the family medicine practice (primary care). For GCM, patients were recruited after a recent discharge from each of the three care settings, with the goal of recruiting all patients within 3 months of that visit. We enrolled participants from these three distinct periods on the care continuum pathway to enable us to capture any potential variation in patient goals and preferences related to current health status. Finally, all patients were required to have moderately- to poorly-controlled DM, defined as follows per setting: presented to the ED with a DM-related problem (acute care), admitted to the hospital for a DM-related problem (post-acute care), or at least 2 measurements of HbA1c > 7.5 in the prior year (primary care). Exclusion criteria included: having a new diagnosis of DM made during that visit; having a significant permanent complication related to DM including end stage renal disease, amputation, or blindness thought related to diabetes; undergoing medical clearance for a detox center or any involuntary court or magistrate order; in police custody or currently incarcerated; or having major communication barriers such as visual or hearing impairment or dementia that would compromise ability to give written informed consent.

For interviews, each setting was treated as a separate study. Thus, we anticipated enrollment of approximately 30 patients per setting, with interviews conducted until thematic saturation was reached and no new ideas were emerging as determined by the interview team [7, 30]. For GCM, we conducted three distinct iterations (A, B, and C), with each iteration including a mix of patients who represented each of the three care settings. There is no standard for how many GCM iterations are needed for “thematic saturation” or complete construct conceptualization. Thus, we conducted three iterations, each of which consisted of three separate sessions, to allow for more complete analysis of the construct under study.

### Patient recruitment and data collection

#### Interviews

Research team members took shifts to screen the electronic medical records (EMR) for potentially eligible patients in the ED. They approached, consented and interviewed eligible patients during or immediately after an ED visit. Team members used auto-generated lists from the EMR to identify potentially eligible post-discharge and primary-care patients. Post-discharge patients were consented and interviewed by phone while primary-care patients were consented and interviewed on site immediately before or after their primary care appointment. We used an open-ended, semi-structured interview guide to discuss outcomes most important to patients when making decisions regarding management of their DM. (see Additional file 1) Interviewers were trained and monitored by a study team member (KLR) who has extensive experience using interviews to elicit patient priorities during acute episodes of care. Interviews were audio recorded, professionally transcribed and coded and analyzed with NVivo 11.0 software [31].

#### Group concept mapping

Team members used EMR-generated lists to identify all potentially eligible patients for GCM. Patients were contacted by phone in a random order to complete screening and assess interest in participation. We aimed for participation of 16–20 patients in each of 3 GCM iterations, and adjusted our target recruitment number after a higher than anticipated participation rate in the first iteration. The GCM process [14, 22, 32] took place over three sessions. In the first session, participants brainstormed ideas in response to the following prompt: “You’re here as a person with diabetes; when people with diabetes seek care, what are they hoping to improve or make happen?” In the second session, participants worked individually to sort the brainstormed ideas into conceptually-similar piles using Concept Systems Global Software [33]. In the final session, participants worked as a group to review the concept maps that were generated by the software based on the results of all the individual sorts, refine the sorting of the ideas, and name each final cluster. A team member (ML) experienced in conducting GCM provided oversight of the entire GCM process and trained all involved research team members. All GCM processes and analysis were conducted using Concept Systems Global Software [33].

Informed consent was performed with all patients prior to the start of the first GCM session. Demographic data were collected for all interview and GCM participants via both self-report and through chart review. Concept mapping participants were compensated $125 for completing all three sessions of a single GCM iteration. Interview participants were compensated $25 upon completion of the interview. Appropriate remuneration was decided by the PAKSAB, with this decision guided primarily by consideration of time and travel requirements of each method.

### Analysis

We compared the processes and outputs of GCM and interviews in two key domains: comprehensiveness and efficiency. Our primary analysis was an assessment of the comprehensiveness of the brainstorming phase of GCM as compared to interviews for content elicitation. Secondary outcomes include an assessment of 1) the comprehensiveness of each method compared to a master list of all content identified across both methods and 2) assessment of the efficiency of interviews compared to both GCM brainstorming and the entire GCM process. For the primary outcome assessment of comprehensiveness, the research team undertook the following process to allow for direct comparison of the PIOs generated by GCM brainstorming and interviews, with all final decisions made by the 3 PAKSAB members working on the research team.

The team used a conventional qualitative content analysis approach to analyze the interview transcripts, with one of the codes being “goals” [34]. All ideas coded to the “goals” node that were in any way relevant to their diabetes care were extracted to create a list of interview-generated PIOs.

For the GCM data, the team first eliminated redundant ideas generated within or between the brainstorming phase of each of the three GCM iterations to generate a “master” list of GCM PIOs. For example, “stop using needles,” “get off insulin or injections,” and “move from insulin to pill” were all thought to convey the same concept, and were collapsed into a single PIO named “eliminate injections.” Each item on the combined list of GCM PIOs was tagged to identify the GCM iteration(s) in which the PIO had been identified.

To compare findings from the 2 methods, the team matched the combined list of GCM PIOs with the interview PIOs. The final matched list of PIOs from both the interviews and GCM was reviewed with the entire PAKSAB to ensure the naming and matching of collapsed PIOs was appropriate. See Table 1 for examples of matched PIOs.

**Table 1:** Examples of Combined Ideas and Final PIO Names

GCM-A	GCM-B	GCM-C	GCM Master PIOs (A + B + C)	Interviews	Final Merged PIOs (GCM + Interview)
Get off insulin; Move from insulin to pill;	Stop using needles; Get faster treatment that doesn’t involve a shot; Stay off insulin	Get off insulin	Eliminate injections	Get off insulin or injections; Avoid insulin or injections	Eliminate injections
Learn from other people with diabetes; Get a peer group of diabetics together to learn from and support each other			Get peer support	Continue or start diabetes group; Be an inspiration to others	Participate in peer support
Eat right	Keep nutrition at recommended guidelines; Get medicine that has bad interactions with unhealthy foods to help you stop eating them	Eat right; Improve self-control to eat appropriate portions	Eat right	Control diet or eat healthy	Eat right
Improve mental health; Improve mood; Manage anxiety and depression caused by diabetes; Understand how to manage anxiety		Have a good spirit; Stay calm; Reduce fear of complications; Reduce fear about having diabetes	Improve mental health	Prevent depression; Be happy; Control mindset or self-control	Improve mental health

For the comparison of the comprehensiveness of the methods, we calculated the percentage of interview PIOs elicited in 1) a single GCM brainstorming iteration (GCM-A) and 2) multiple GCM brainstorming iterations (GCM A + B + C). We also assessed the overall comprehensiveness of both methods by calculating the percentage of total merged PIOs (GCM + interviews) elicited in 3) interviews, 4) GCM-A and 5) GCM A + B + C. (Table 2).

**Table 2:** Comparisons of Comprehensiveness of GCM and Interviews

Comparison	GCM-A PIOs	GCM (A + B + C) PIOs	Interview PIOs	Final Merged PIOs (GCM + Interview)
1	X		X	
2		X	X	
3			X	X
4	X			X
5		X		X

For the secondary outcome of the efficiency of each method, we developed a list of the primary tasks required by each type of participant (patient and research team members) for each method (interviews, GCM brainstorming, and GCM entire process). The research team estimated the average length of time for each task by reviewing records in the team activity log. Team-member variables were calculated based on working with a research team of 3 individuals. Average travel time was calculated for patient participants and research team members based on the distance traveled. For the cost outcomes, we included what were thought to be necessary financial costs associated with each method. We include both fixed costs (software) and variable costs (participant remuneration and transcription). Certain additional costs, such as fees to rent specific rooms on campus where GCM was performed, were omitted as these were determined by the team to be highly discretionary. For interviews, we report the efficiency to conduct interviews in a single setting to saturation (*n* = 30). While we conducted more interviews (*n* = 89) in this study, this was because of unique goals specific to our study that necessitated conducting interviews to saturation in each of three different healthcare settings. As the majority of interview studies are performed with a single population to thematic saturation, we chose to provide efficiency data for a single set of interviews to saturation. In addition, though our primary comprehensiveness comparison was limited to interviews and GCM brainstorming, we include data on the entire GCM process within the efficiency analysis to provide researchers with more robust data on which to inform future research design decisions. For situations in which researchers decide to use GCM brainstorming as part of the entire GCM process, this information on the efficiency of the entire method will be important for research planning purposes.

We compared participants in each GCM iteration to each other. We also compared the combined GCM participants to interview participants. We used chi-square for categorical variables and *t*-tests for continuous variables. We report descriptive statistics to describe the overall population enrolled in each method.

## Results

### Study population and characteristics

Our final study population included 89 interview participants and 52 total GCM participants (24 in session A, 14 in session B and 14 in session C). There were no statistically significant differences between participants in each of the GCM iterations. When comparing GCM participants to interview participants, GCM participants were significantly more likely to be Black and to have a high school education or less compared to interview participants. All of the patient participants had moderately- to poorly-controlled DM, with mean HbA1c of 10.2 and 9.2 (interviews and GCM, respectively). See Table 3 for participant demographics.

**Table 3:** Participant Demographics for Interviews and Group Concept Mapping (GCM)^a^

	Interviews, *N* = 89	GCM, *N* = 52
Age, mean (range), SD	54.6 (23–88), 13.8	55.6 (23–95), 14.7
Ethnicity		
Hispanic/Latino	8 (9)	3 (6)
Not Hispanic/Latino	80 (90)	49 (94)
Race		
White	24 (27)	5 (10)
Black	60 (68)	42 (81)
Other	4 (5)	4 (9)
Sex		
Male	40 (45)	26 (50)
Female	49 (55)	24 (46)
HbA1c – mean (range), SD	10.2 (5.6–27.0), 3.3	9.2 (5.3–14.8), 2.6
Body Mass Index – mean (range), SD	34.8 (11–73.5), 10.3	34.4 (21.9–60.4), 8.8
Hospital Admits – mean, SD	2.3, 4.1	1.5, 1.8
ED Visit - mean, SD	2.8, 4.3	2.5, 2.7
Doctor Visits - mean, SD	11.2, 4.3	8.3, 9.1
Education		
Less than High School	4 (5)	8 (15)
High school graduate	68 (76)	29 (56)
College Degree	4 (5)	12 (23)
Post-Grad degree	13 (15)	3 (6)
Income		
< 10 K	15 (21)	7 (14)
10-25 K	22 (31)	22 (42)
25-50 K	19 (27)	14 (27)
50-99 K	7 (10)	3 (6)
> 100 K	8 (11)	2 (4)
Years Since Diagnosis		
< 1 year	2 (2)	2 (4)
1–5 years	12 (13)	15 (29)
> 5 years	74 (83)	33 (64)
Health status (mean, SD) (range 1–5,: 1 = excellent and 5 = poor)	3.6, 0.9	3.6, 0.8

^a^All variables were self-report, aside from A1c and Body Mass Index. Some percent totals do not equal 100 due to missing data

### Comprehensiveness of interviews and group concept mapping brainstorming

We identified 26 PIOs in interviews, 33 in GCM-A, and 38 across all three GCM iterations. GCM-A identified 77% (20/26) and GCM A + B + C identified 88% (23/26) of PIOs that were identified in interviews. When comparing both GCM and interviews to all PIOs (interviews + GCM), GCM A identified 80% (33/41), GCM A + B + C identified 93% (38/41) and interviews identified 63% (26/41) of all PIOs. (Table 4).

**Table 4:** Patient-Important Outcomes (PIOs) Generated By Each Method

	Interviews	GCM-A	GCM A + B	GCM A + B + C	All PIOs^a^
Total # of PIOs generated	26	33	36	38	41
Number of interview PIOs identified by GCM	–	20	21	23	–

^a^Includes all PIOs identified in interviews and GCM

### Efficiency of interview and group concept mapping

Table 5 provides a detailed log of the time and cost of interviews, GCM brainstorming and the entire process of GCM. Data are provided for performing 30 interviews (one care setting), a single GCM brainstorming session, and a single full iteration of GCM (includes all 3 sessions: brainstorming, sorting & rating, and interpretation). GCM brainstorming required 2 more hours of patient-participant time than interviews (3 vs 1 h), yet it required the least research team time of all methods (78 h) and had the lowest financial cost ($1200). A full iteration of GCM compared to interviews required 7 more hours per patient participant (8 vs 1 h), took 191 h less of the research team time (104 vs 295 h), and cost $1870 more ($5000 vs $3130).

**Table 5:** Resources Utilized per Patient Engagement Method

		Interviews^b^ (one setting)	GCM Brainstorming (one iteration)	GCM^c^ (one iteration)
Patient time (*hours/person)*	Travel	0	1	2
	Participation	1	2	6
	Total hours	1	3	8
Research team time^a^ (hours/team)	Training	20	3	11
	Patient recruitment	66	59	59
	Travel	60	6	12
	Conducting method	38	8	18
	Analysis	133	2	4
	Total hours	295	78	104
Variable Costs	Patient incentives	$750	$1200	$3000
	Transcription	$1000	n/a	n/a
Fixed Costs	Data Analysis Software	$1380	n/a	$2000
	Total cost	3130	1200	5000
Qualities about data collected	Type of data collected	List of PIOs with detailed patient perspective/context	List of PIOs without context	List of PIOs without context though sorted into overarching themes
	Minimum personnel needed	Investigator & Patient Advocate	Investigator & Patient Advocate	Investigator, RA, Patient Advocate
	Resources for Analysis	Qualitative Analysis Software	Patient Advisory Board	Concept Mapping Software
	Level of burden	Low on patient, high on researchers	Low on patient and researchers	High on patient, medium on researchers

^a^Research team of 3 people, ^b^30 interviews in one setting, ^c^24 participants in one iteration

## Discussion

In our comparison of the comprehensiveness of GCM brainstorming and interviews for eliciting PIOs related to diabetes care, we found that a single session of GCM brainstorming elicited 77% of PIOs identified across 89 interviews, and three sessions of GCM brainstorming elicited 88% of interview PIOs. In addition, a single session of GCM brainstorming identified more unique PIOs overall than interviews. These findings suggest that GCM brainstorming is more comprehensive than interviews for content elicitation. Regarding efficiency of each method, we found that for solely conducting content elicitation, GCM brainstorming is more efficient than interviews for both time and financial costs. For more extensive analyses in which the full GCM process is conducted, GCM is requires less researcher time, more per person patient time, and higher financial cost. Exploration is warranted regarding when each of these methods might be used, and context for when GCM in its entirety would be beneficial.

While there are no prior studies to our knowledge comparing the methods of GCM and interviews across the domains of both comprehensiveness and efficiency, there has been some prior work comparing the process or product of different methods of patient engagement [6, 24, 35–37]. Two studies compared interviews and focus groups for eliciting patient perspective related to their chronic disease and had conflicting results. Coenen et al. concluded that focus groups are more time consuming yet more comprehensive than interviews [36], while Rat et al. concluded that individual interviews were more comprehensive than focus groups [6]. Another study assessed the effectiveness and efficiency of GCM by comparing the results produced by GCM to those from a prior meta-analysis, concluding that concept mapping was effective and efficient [35]. Finally, Humphrey et al. compared interviews, concept mapping, and social media review to identify symptoms related to ankylosing spondylitis, and concluded that interviews were most time intensive though provided the greatest conceptualization of the patient experience, while GCM adds value when there is greater need to minimize researcher bias within the research topic [24].

Our findings expand prior work by providing information on both the process and outcomes of GCM and interviews for engaging patients to identify priorities related to their treatment. We suggest that the selection of the optimal method for engaging patients depends on a number of factors including the level of detail of patient input required, expertise of the research team, financial resources and amount of time available, population to be engaged, and appropriateness of the research question for group discussion. GCM in its entirety provides organization and a framework in which to understand ideas that are generated during the initial brainstorming phases. Participant-generated maps of the brainstormed ideas suggest how the PIOs interact with each other, and participant rating can help in selecting priority PIOs for use in research. This higher-level conceptualization is beyond that provided by methods such as brainstorming and interviews, and thus researchers may choose to use GCM when needs extend beyond simple content generation. Yet GCM is time intensive for patients and has potentially high costs including software and participant incentives related to the high time requirements. It also requires certain cognitive and technical abilities (i.e. understand conceptually how to sort ideas into piles of similarity, use a computer and mouse, be able to perform rating tasks) that we found challenged many of our study participants and may limit its application in some settings. Interviews, in contrast, may provide greater information regarding lived experience and other context important to PIOs, and can be tailored to the individual needs of each patient. As discussed above, however, interviews have a high time burden on researchers, and involve extensive research team analysis, which has potential to inject significant bias. Finally, brainstorming alone lacks addition of the other contextual elements provided by GCM or interviews, yet brainstorming is the most efficient method and it has very few participant or researcher barriers.

Our PAKSAB was a vital component of this work. PAKSAB members conducted analysis of the brainstorming data, including merging and naming of similar ideas, to generate the final GCM PIOs. They then performed the matching of GCM and interview PIOs, thus eliminating researcher bias from these key analytic phases. By working with our patient advisory board, we were able to combine ideas from multiple brainstorming sessions to produce one final list of PIOs and completed analysis much more efficiently than with conducting full GCM iterations. Thus, based on our experience, we suggest the combination of group brainstorming for content elicitation along with sorting and rating by a patient advisory board to identify higher level conceptual domains is a comprehensive and efficient method to identify PIOs.

### Limitations

We conducted this study within a specific population: patients with moderately- to poorly-controlled DM who all seek care within the same health system. We are unable to assess whether results are generalizable across other populations, and it is possible that individuals with different healthcare experiences and needs, or with varying demographics (such as higher overall education level), would engage differently in both methods. While we strove to enroll a similar population in both methods and used the same eligibility criteria, demographics did vary between groups. Although GCM participants had overall lower education level, GCM brainstorming was actually more comprehensive than interviews, suggesting that low education was not a barrier to participation. It is possible that different methods are inherently more acceptable to different demographic groups, which will need exploration in future work. In addition to demographic differences between groups, the timing of the methods resulted in enrolling patients who were at different stages of illness, thus potentially varying the goals identified in each method. As our goal was to identify patient goals related to seeking care, the most relevant time to perform interviews was during a care visit. It is not possible to perform GCM with a group of patients who are all currently receiving care in the ED or at a primary care visit, and thus the different timing of engagement was a built-in factor to this study. As such, it is an important part of our findings more than a limitation to conclude that patients who were engaged after an acute stage of illness (GCM) still identified the majority of priorites as those engaged during the actual illness (interviews). This finding also warrants further study, as expanding the time window to be able to engage patients outside of immediate healthcare encounters to identify goals and needs potentially reduces both patient and researcher burden.

Our assessment of efficiency was impacted by factors specific to our setting, such as how far patients and the research team live and how much we provided for patient incentives, as well as what our team decided was important to include. For example, costs such as qualitative analysis software may not be relevant to those who already have a software license, and training time will differ based on prior team experience. While time commitment to conduct the project from both patients and researchers is probably the most stable figure in this assessment, we have included detail regarding other expenses that our team thought important to consider to maximize utility of this assessment, listing them separately to allow for individual interpretation and modification. Of note, we did not incorporate consideration of logistic needs specific to GCM, such as coordination of access to computers and a room large enough to accommodate all the participants in a GCM session.

In addition, although we randomly sampled patients from those identified as potentially eligible, our sampling produced a convenience sample and thus potentially introduced bias as more engaged and motivated patients likely enrolled in the study. This bias is unavoidable, but should be balanced across the groups, and should have produced a sample bias consistent with any convenience sample of patients. Finally, the same research team conducted all the interviews and the three GCM sessions, thus findings may vary across other patient populations, in other settings, and for other research groups. Regardless of potential variation, these results are valuable to inform researchers of considerations regarding when to use interviews versus GCM or other group engagement forums for eliciting patient-centered outcomes.

## Conclusion

We conclude that the brainstorming phase of GCM is more comprehensive than individual interviews as a means of content elicitation for identifying PIOs related to seeking care for use in research and clinical practice. There are benefits to interviews, such as depth and the ability to conduct them at the convenience of each individual patient, and full GCM iterations, such as higher level conceptualization of patient ideas, that should drive their use in certain scenarios. For many situations, however, group brainstorming is an accessible and powerful tool that can empower researchers to identify priority patient-centered outcomes for use in research.

## Additional file

Additional file 1: Interview guide. Semi-structured interview guide used to conduct all the interviews reported within this manuscript. (DOCX 33 kb)

## Abbreviations

EMR: Electronic medical records
GCM: Group concept mapping
PAKSAB: Patient Advocate and Key Stakeholders Advisory Board
PIOs: Patient-important outcomes
PROs: Patient-reported outcomes
