# Supplementary material for: The power of the group: comparison of interviews and group concept mapping for identifying patient-important outcomes of care
Source: BMC Med Res Methodol. 2019 Jan 8;19:7. doi: 10.1186/s12874-018-0656-x (PMC6323717; doi:10.1186/s12874-018-0656-x)
Supplement: Supplementary file 1 — Interview guide. Semi-structured interview guide used to conduct all the interviews reported within this manuscript. (DOCX 33 kb) [file 12874_2018_656_MOESM1_ESM.docx]

**Additional File 1: Interview Guide**

Thank you for agreeing to participate in this research study. In the past, medical research has often focused on asking doctors what they think is most important in improving health care for their patients, but we have realized that patients may have different priorities and objectives when they seek health care. Sometimes, patients and doctors also think about different things when deciding if a treatment is successful. Today we would like to know what things *you* think about when deciding whether your diabetes treatment has been successful. It can be *anything* you consider important. We want to hear about any little things that matter to you, as well as anything you want to happen in the long term.

We hope to use this information to improve the delivery of health care and prioritize the concerns patients tell us are important to them.

Do you have any questions or concerns before we begin?

THIS IS AN INTERVIEW WITH STUDY ID _____ BY INTERVIEWER _________ ON _____________.

1. I’d like to learn a little bit about you to start. Tell me about yourself?
2. What are some things that are important to you in your life?
3. What is it like living with diabetes?
   1. How would your life be different if you didn’t have diabetes?
4. What do you think diabetes is doing to your body?
   1. Why do you think you have diabetes?
5. When thinking about your diabetes, what worries or concerns do you have?
6. What do you do to manage your diabetes (when you’re not seeing your doctor)?
7. How well managed do you think your diabetes is?
   1. What things do you think about when deciding whether your diabetes management has been successful?
   2. What things about your diabetes management are working well?
   3. What things are not working as well?
8. What things can get in the way of you taking care of your diabetes?

1. If you didn’t have access to any bloodwork or other test results, how would you tell if your diabetes is well managed?
2. Who are you comfortable talking with about your diabetes.
   1. What do you normally talk about?
3. Who do you go to for your diabetes care? – Tell me about your relationship with XXX.
4. What motivates you to visit your doctor?
   1. What things in your life are you hoping to change or improve?
   2. What things are you hoping to prevent or avoid?
   3. What are your goals for your treatment?
5. What things do you think your doctor uses right now to say how well your diabetes is controlled?
   1. What things do you wish your doctor would think about?
6. Tell me about a time when you didn’t follow your doctor’s recommendations for your diabetes treatment.
7. Select from the appropriate setting
   1. Emergency Department: What is the primary reason for your medical visit today?
   2. Primary Care: What is the primary reason for your medical visit today?
   3. Post-Acute: What was the primary reason for your medical visit when you were hospitalized?
8. Select from the appropriate setting
   1. Emergency Department: Did you talk about your diabetes management at your visit today?
   2. Primary Care: Did you talk about your diabetes management at your visit today?
   3. Post-Acute: Did you talk about your diabetes management when you were hospitalized?
9. It sounds like xxx are important to you for your diabetes management… ASK BOTH ?S
   1. Am I missing anything?
   2. Which one or two are most important to you? (*xxx to be filled in by interviewer based on what the patients have responded to the above questions*)

1. Is there anything else you’d like to add, that I may not have asked about?
